# Autism-Linked Mutations in α_2_δ-1 and α_2_δ-3 Reduce Protein Membrane Expression but Affect Neither Calcium Channels nor Trans-Synaptic Signaling

**DOI:** 10.3390/ph17121608

**Published:** 2024-11-28

**Authors:** Sabrin Haddad, Manuel Hessenberger, Cornelia Ablinger, Clarissa Eibl, Ruslan Stanika, Marta Campiglio, Gerald J. Obermair

**Affiliations:** 1 Division of Physiology, Department of Pharmacology, Physiology, and Microbiology, Karl Landsteiner University of Health Sciences, 3500 Krems, Austria; sabrin.haddad@kl.ac.at (S.H.);; 2 Institute of Physiology, Medical University Innsbruck, 6020 Innsbruck, Austria

**Keywords:** voltage-gated calcium channels, calcium current, trans-synaptic function, auxiliary subunit, autism spectrum disorder, electrophysiology, cultured hippocampal neurons

## Abstract

Background: α_2_δ proteins regulate membrane trafficking and biophysical properties of voltage-gated calcium channels. Moreover, they modulate axonal wiring, synapse formation, and trans-synaptic signaling. Several rare missense variants in CACNA2D1 (coding for α_2_δ-1) and CACNA2D3 (coding for α_2_δ-3) genes were identified in patients with autism spectrum disorder (ASD). However, the pathogenicity of these variants is not known, and the molecular mechanism by which α_2_δ proteins may contribute to the pathophysiology of autism is, as of today, not understood. Therefore, in this study we functionally characterized two heterozygous missense variants in α_2_δ-1 (p.R351T) and α_2_δ-3 (p.A275T), previously identified in patients with ASD. Methods: Electrophysiological recordings in transfected tsA201 cells were used to study specific channel-dependent functions of mutated α_2_δ proteins. Membrane expression, presynaptic targeting, and trans-synaptic signaling of mutated α_2_δ proteins were studied upon expression in murine cultured hippocampal neurons. Results: Homologous expression of both mutated α_2_δ proteins revealed a strongly reduced membrane expression and synaptic localization compared to the corresponding wild type α_2_δ proteins. Moreover, the A275T mutation in α_2_δ-3 resulted in an altered glycosylation pattern upon heterologous expression. However, neither of the mutations compromised the biophysical properties of postsynaptic L-type (Ca_V_1.2 and Ca_V_1.3) and presynaptic P/Q-type (Ca_V_2.1) channels when co-expressed in tsA201 cells. Furthermore, presynaptic expression of p.R351T in the α_2_δ-1 splice variant lacking exon 23 did not affect trans-synaptic signaling to postsynaptic GABA_A_ receptors. Conclusions: Our data provide evidence that the pathophysiological mechanisms of ASD-causing mutations of α_2_δ proteins may not involve their classical channel-dependent and trans-synaptic functions. Alternatively, these mutations may induce subtle changes in synapse formation or neuronal network function, highlighting the need for future α_2_δ protein-linked disease models.

## 1. Introduction

Autism spectrum disorder (ASD) comprises a heterogeneous group of early-onset neurodevelopmental conditions defined by impaired social interaction and repetitive, restrictive behaviors [1,2]. Meta-analysis correlations of monozygotic twins strongly suggest genetic factors accounting for the etiologies of ASDs [3]. Large-scale whole-exome sequencing studies identified more than 100 risk genes for ASDs, which are categorized into two major functional groups: genes involved in transcriptional/epigenetic regulation (chromatin regulators and transcription factors) and genes involved in neuronal connectivity (synapse formation and synaptic transmission) [4,5]. Neuronal α_2_δ-1 and α_2_δ-3 proteins fall into the latter group [4,6]. They are encoded by CACNA2D1 and CACNA2D3 genes, respectively, serve as auxiliary subunits of voltage-gated calcium channels, and are critical regulators of axonal wiring, synapse formation, and synaptic transmission [7,8].

Voltage-gated calcium channels (VGCCs, or Ca_V_s) control the entry of calcium into cells and thereby regulate a variety of vital neurophysiological functions, including neurotransmitter release, synaptic plasticity, and gene transcription [9]. Neuronal VGCCs are multimeric complexes associated with β and α_2_δ auxiliary subunits [10]. The α_2_δ subunits are membrane-attached, extracellular proteins encoded by four genes, giving rise to four isoforms (α_2_δ-1 to -4), out of which three isoforms (α_2_δ-1 to -3) are highly expressed throughout the central nervous system [11]. Besides their roles as regulators of membrane expression and biophysical properties of VGCCs, α_2_δ proteins have emerged as important modulators of synapse formation and synaptic plasticity [7,8,12,13,14,15]. Therefore, the association of CACNA2D genes with neurodevelopmental disorders, including ASDs, is not surprising [13].

Mice lacking α_2_δ-3 show increased anxiety-like behavior [16,17] and defective object-based memory [17], indicating a strong association between the CACNA2D3 gene and autism. α_2_δ-3 knockout mice also display sensory cross-modal activation (synesthesia) [18], a neurological condition in which a stimulus in one sensory modality (in this case thermal pain) triggers perception of another unstimulated modality (brain regions involved in vision, olfaction, and hearing). Intriguingly, two studies revealed a significant increase in synesthesia prevalence in patients with ASD [19,20], strengthening the link of CACNA2D3 to ASD. Furthermore, a recent study links conditional CACNA2D3 knockout in parvalbumin-expressing cortical interneurons to autism-like behavior [21]. Besides CACNA2D3, the human CACNA2D1 gene has also recently been associated with neurodevelopmental disorders, including intellectual disability, ASD, and developmental and epileptic encephalopathy (DEE) [22,23,24], illustrating the essential role and unique functions of α_2_δ-1 along with other α_2_δ proteins in the proper development of neuronal networks. Furthermore, both CACNA2D1 and CACNA2D3 genes are included in the Simons Foundation Autism Research Initiative (SFARI) gene list, which serves as an extensive database that includes all genes associated with autism risk [25].

Several potential autism-causing mutations in the coding sequence of CACNA2D1 and CACNA2D3 have been identified in patients with ASD (summarized in Table 1). However, these variants were discovered through large-scale whole-exome or -genome sequencing. Consequently, their effects on protein function are largely unstudied, and hence the assessment of their pathogenicity is not clear-cut. Therefore, in our study we aimed to characterize the structural and functional consequences of two potential autism-causing missense mutations: a de novo p.R351T mutation in α_2_δ-1 found by exome sequencing in a family of the Simon simplex collection (SSC) of ASDs [26] and a paternally inherited p.A275T mutation in α_2_δ-3 identified in a Chinese ASD cohort [27]. Both mutations reduce the membrane expression of α_2_δ proteins without affecting total protein levels. While α_2_δ-3_A275T proteins show a modified glycosylation pattern, the mutations disrupted neither calcium channel trafficking and function, nor trans-synaptic signaling.

## 2. Results

### 2.1. Arginine 351 and Alanine 275 Are Evolutionary Conserved Amino Acids with Key Predicted Roles in Stabilizing the Protein Structure of α_2_δ-1 and α_2_δ-3, Respectively

**Figure 1:**
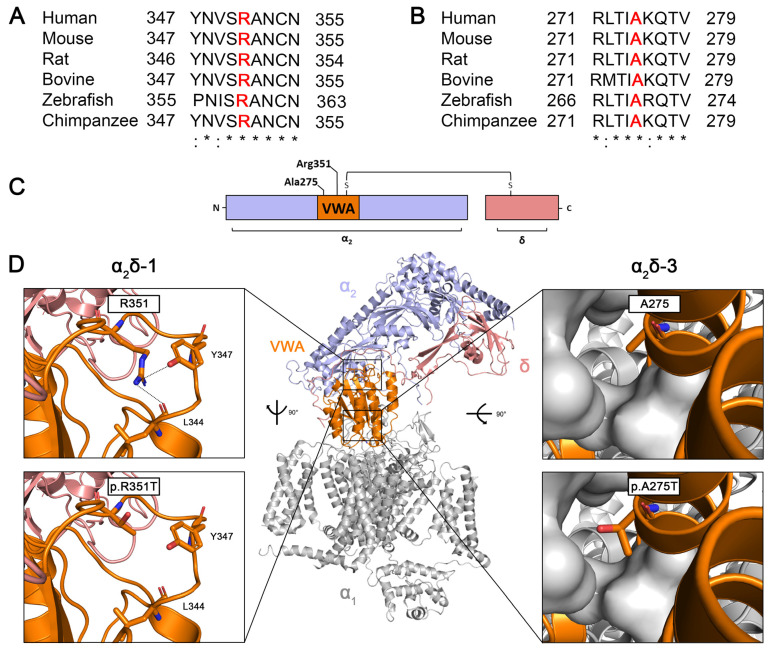
**The highly conserved R351 (α_2_δ-1) and A275 (α_2_δ-3) residues are predicted to be important for the integrity of the mature protein**. Amino acid sequence alignments of (**A**) α_2_δ-1 and (**B**) α_2_δ-3 between different species. Positions corresponding to arginine 351 (R351) and alanine 275 (A275) in human α_2_δ-1 and α_2_δ-3, respectively, are highlighted in bold red. (**C**) Schematic overview of α_2_δ protein illustrating the positions of the R351 and A275 residues within the VWA domain. (**D**) Cryo-EM structure of human α_2_δ-1 protein (color code as in **C**) in complex with the human Ca_V_2.1 channel (gray, PDB code: 7MIY). On the left panel, the enlarged image shows the ionic interactions (dashed lines) of R351 with tyrosine 347 (Y347) and leucine 344 (L344) (upper image), which stabilize the loop. Substituting R351 with threonine prevents the formation of these stabilizing interactions (lower image). On the right panel, the enlarged image of the VWA domain of α_2_δ-3 suggests that A275 participates in a hydrophobic pocket with surrounding residues in the VWA domain (gray, surface representation) to stabilize the fold of the VWA domain (upper image). Substituting A275 with threonine, an amino acid with a polar side chain, may disrupt the hydrophobic pocket (lower image). (Color code: α_2_ peptide in purple, VWA domain in orange, δ peptide in pink, and α_1_ subunit in gray). AlphaFold per-residue model confidence score (pLDDT) for A275 is very high with a value of 94.1.

CACNA2D genes encode single precursor proteins that are post-translationally cleaved into disulfide-bonded α_2_ and δ polypeptides. Amino acid sequence alignments between different species show that human arginine 351 (R351) in α_2_δ-1 (Figure 1A) and alanine 275 (A275) in α_2_δ-3 (Figure 1B) are evolutionarily conserved amino acids. R351 and A275 are located within the von Willebrand factor type A domain (VWA) of the metal ion-dependent adhesion site (MIDAS) (Figure 1C), which has been previously shown to play a key role in trafficking calcium channels to the plasma membrane [37]. The Cryo-EM structure of α_2_δ-1 (pdb: 7miy; Figure 1D, upper left panel) shows that R351 is anchored within a cleft by a strong cation–π interaction with tyrosine 347 (Y347) and additional ionic interactions involving Y347 and leucine 344 (L344). The p.R351T mutation disrupts these interactions (Figure 1D, lower left panel), destabilizing the loop within the VWA domain of the α_2_δ-1 protein and thereby affecting its overall structure. Moreover, the AlphaFold model of α_2_δ-3 suggests that A275 is critical for stabilizing the helices within the VWA domain by contributing to a hydrophobic pocket (Figure 1D, upper right panel). Substituting alanine, a hydrophobic amino acid, with threonine, a polar amino acid, may disrupt this hydrophobic pocket, potentially affecting the overall structure of the VWA domain (Figure 1D, lower right panel). Therefore, we hypothesize that p.R351T in α_2_δ-1, and p.A275T in α_2_δ-3 affect the structural and functional integrity of α_2_δ proteins.

### 2.2. Reduced Membrane Expression of α_2_δ-1_R351T and α_2_δ-3_A275T Variants in Differentiated Cultured Hippocampal Neurons

**Figure 2:**
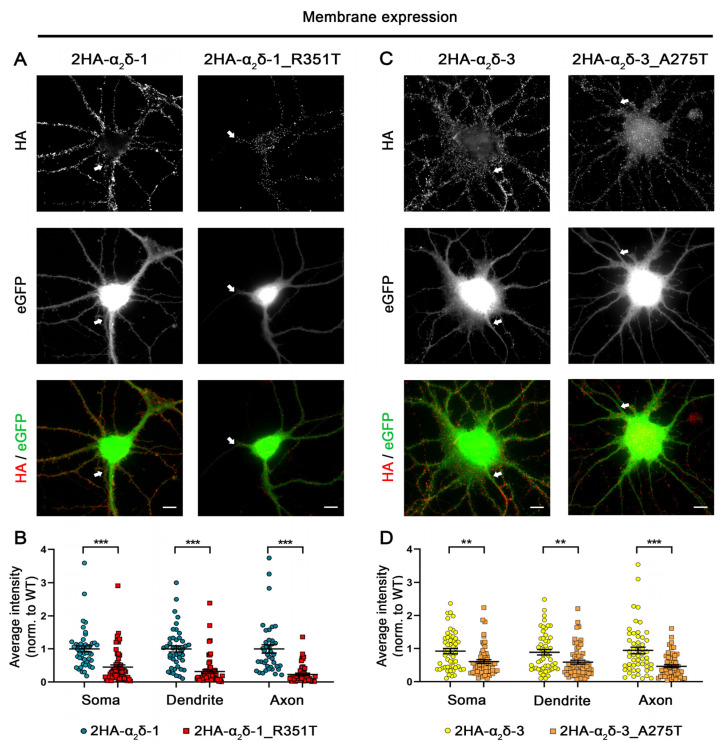
**Strongly reduced membrane expression of α_2_δ-1_R351T and α_2_δ-3_A275T in differentiated cultured hippocampal neurons**. (**A**,**C**) Representative images of primary cultured hippocampal neurons (DIV 21) transfected with soluble eGFP together with either HA-tagged WT (2HA-α_2_δ-1 or 2HA-α_2_δ-3) or mutated (2HA-α_2_δ-1_R351T or 2HA-α_2_δ-3_A275T) α_2_δ. Anti-HA live-cell labelling demonstrates a reduced staining intensity of both α_2_δ-1_R351T and α_2_δ-3_A275T in the soma, dendrites, and axons (indicated by arrows) compared to the respective WT α_2_δ. (**B**,**D**) Quantification of the average HA fluorescent intensities in the different compartments shows a reduced surface expression of mutated α_2_δ compared to WT α_2_δ. Graphs show values for individual cells (dots) and means ± SEM (lines). All values were normalized to the mean fluorescence intensity of the WT 2HA-α_2_δ within each culture preparation. **Statistics**: (**B**) Data were obtained from three independent culture preparations; 45 and 57 cells expressing WT or mutated HA-tagged α_2_δ-1 were analyzed, respectively. Unpaired *t* test, soma: t_(100)_ = 4.5; *** *p* < 0.0001, dendrite: t_(100)_ = 6.5; *** *p* < 0.0001, axon: t_(100)_ = 6.3; *** *p* < 0.0001. (**D**) Data were obtained from four independent culture preparations; 52 and 60 cells expressing WT or mutated HA-tagged α_2_δ-3 were analyzed, respectively. Unpaired *t* test, soma: t_(110)_ = 3.3; ** *p* = 0.0013, dendrite: t_(110)_ = 2.9; ** *p* = 0.0035, axon: t_(110)_ = 4.6; *** *p* < 0.0001. Scale bars, 10 µm.

The predicted consequences of the mutations may influence the folding of α_2_δ protein, potentially leading to altered forward trafficking or a reduction in the stability of α_2_δ expression at the plasma membrane. Therefore, we first studied the consequences of these two potential autism-causing mutations on the protein membrane expression. To this end, HA-epitope-tagged wild-type (WT) or mutated α_2_δ proteins were expressed together with soluble eGFP in mouse cultured hippocampal neurons. In comparison to WT α_2_δ-1, live-cell immunostaining revealed that surface expression of α_2_δ-1_R351T was strongly reduced in all three main neuronal compartments, the soma, dendrites, and axons (45% in soma, 31% in dendrites, and 23% in axons compared to WT α_2_δ-1) (Figure 2A,B). Similarly, although less pronounced, α_2_δ-3_A275T showed a reduced membrane expression compared to WT α_2_δ-3 (66% in soma, 66% in dendrites, and 52% in axons compared to WT α_2_δ-3) (Figure 2C,D).

**Figure 3:**
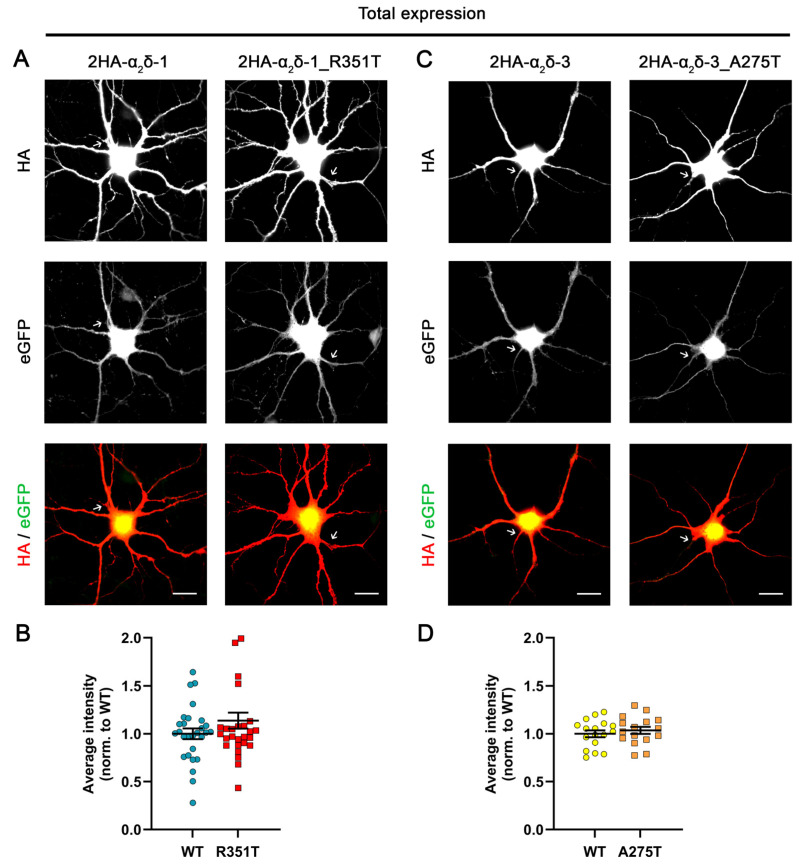
**Comparable overall expression levels of mutated and corresponding WT α_2_δ proteins.** Primary cultured hippocampal neurons were transfected with soluble eGFP together with either HA-tagged WT or mutated α_2_δ. (**A**,**C**) Representative images of permeabilized anti-HA immunostaining, eGFP fluorescence, and anti-HA/eGFP overlay. Axons are indicated by arrows. (**B**,**D**) Analysis of whole-cell fluorescence intensities. Values for individual cells (dots) and means ± SEM (lines). All values were normalized to the mean fluorescence intensity of the WT 2HA-α_2_δ within each culture preparation. **Statistics**: (**B**) Data were obtained from three independent culture preparations; 28 cells expressing WT or mutated HA-tagged α_2_δ-1 were analyzed. Unpaired *t* test, t_(54)_ = 1.38; *p* = 0.17. (**D**) Data were obtained from two independent culture preparations; 17 and 16 cells expressing WT or mutated HA-tagged α_2_δ-3 were analyzed, respectively. Unpaired t test, t_(31)_ = 0.7; *p* = 0.49. Scale bars, 20 µm.

Following translation, missense mutations may disrupt protein folding, resulting in protein degradation. Therefore, the reduced membrane expression of the mutated protein could be attributed to lower overall protein levels, rather than to impaired forward trafficking or an increased rate of internalization. To test this hypothesis, we performed permeabilized anti-HA staining and analyzed whole-cell fluorescence intensity in transfected neurons (Figure 3). Quantifying the overall immunofluorescence signals shows a comparable average immunolabeling intensity between mutant α_2_δ proteins and the corresponding WT proteins (Figure 3B,D), implying that the mutations do not affect the total expression levels of α_2_δ proteins. Taken together, these data show that both p.A275T and p.R351T reduce the membrane expression of α_2_δ-3 and α_2_δ-1, respectively, compared to the corresponding WT α_2_δ proteins, without affecting the overall expression level.

### 2.3. Reduced Membrane Expression but Unaltered Overall Protein Expression of α_2_δ-1_R351T upon Heterologous Expression

**Figure 4:**
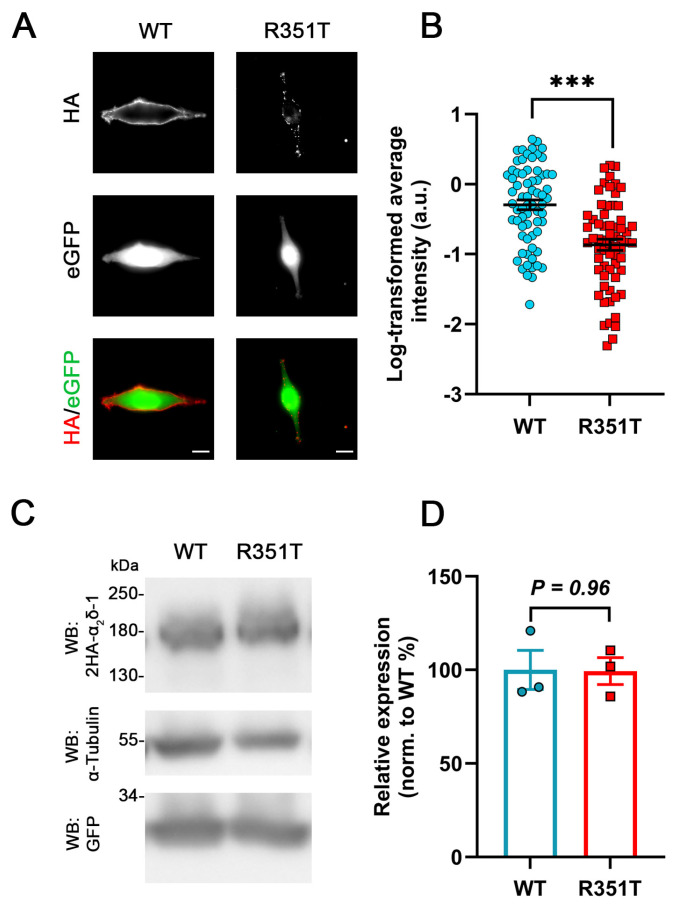
**Strongly reduced membrane expression of α_2_δ-1_R351T in tsA201 cells.** tsA201 cells were co-transfected with soluble eGFP together with either WT or mutated α_2_δ-1 (R351T), both of which are tagged with double HA tag at the N-terminus. (**A**) Representative images of anti-HA live-cell-labelled tsA201 cells. The membrane localization of 2HA-α_2_δ-1 (WT) is characterized by a smooth, fine-dotted pattern on the surface of tsA201 cells. In contrast, the labelling of 2HA-α_2_δ-1_R351T (R351T) exhibits a sparsely dotted localization pattern, accompanied by a reduced overall fluorescence intensity. (**B**) Quantification of the membrane expression of WT and mutated α_2_δ-1. Log-transformed anti-HA live-cell staining intensities (arbitrary units) are shown for individual cells (dots) and means ± SEM (lines). Data were obtained from three independent experiments, and 68 and 64 cells transfected with WT or mutated HA-tagged α_2_δ-1 were analyzed, respectively. (**C**) Immunoblot of whole-cell lysates obtained from tsA201 cells transfected with 2HA-α_2_δ-1 (left lane) or 2HA-α_2_δ-1_R351T (right lane). α_2_δ-1 was detected with an anti-HA antibody (upper panel), anti-α-tubulin labelling was used as a loading control (middle panel), and anti-GFP labelling was used for comparing the transfection efficiency (lower panel). (**D**) Quantification of protein expression levels of α_2_δ relative to GFP expression and normalized to WT expression. **Statistics**: (**B**) unpaired two-tailed *t*-test, t_(130)_ = 5.5; *** *p* < 0.0001; (**D**) unpaired two-tailed *t*-test, t_(4)_ = 0.05; *p* = 0.96. Scale bars, 10 μm.

Because membrane expression of α_2_δ-1_R351T in hippocampal neurons was strongly reduced, we next studied the membrane expression of α_2_δ-1_R351T upon heterologous expression. HA-epitope-tagged WT or mutated α_2_δ-1 were expressed together with soluble eGFP in tsA201 cells. Anti-HA live-cell immunostaining revealed that, similar to homologous expression, α_2_δ-1_R351T showed a strongly reduced surface expression compared to WT α_2_δ-1 (Figure 4A,B). Moreover, western blot (WB) analysis revealed that, similar to homologous expression, the overall protein levels were comparable between tsA201 cells transfected with WT or mutated α_2_δ-1 (Figure 4C,D), confirming that p.R351T does not alter the total expression level of α_2_δ-1. Together, these data show that the R351T mutation similarly affects membrane trafficking of α_2_δ proteins in heterologous and homologous cells, supporting the use of heterologous expression in tsA201 for analyzing the consequences of mutated α_2_δ proteins on calcium channel physiology.

### 2.4. The p.A275T Mutation Alters the Glycosylation Pattern of α_2_δ-3

**Figure 5:**
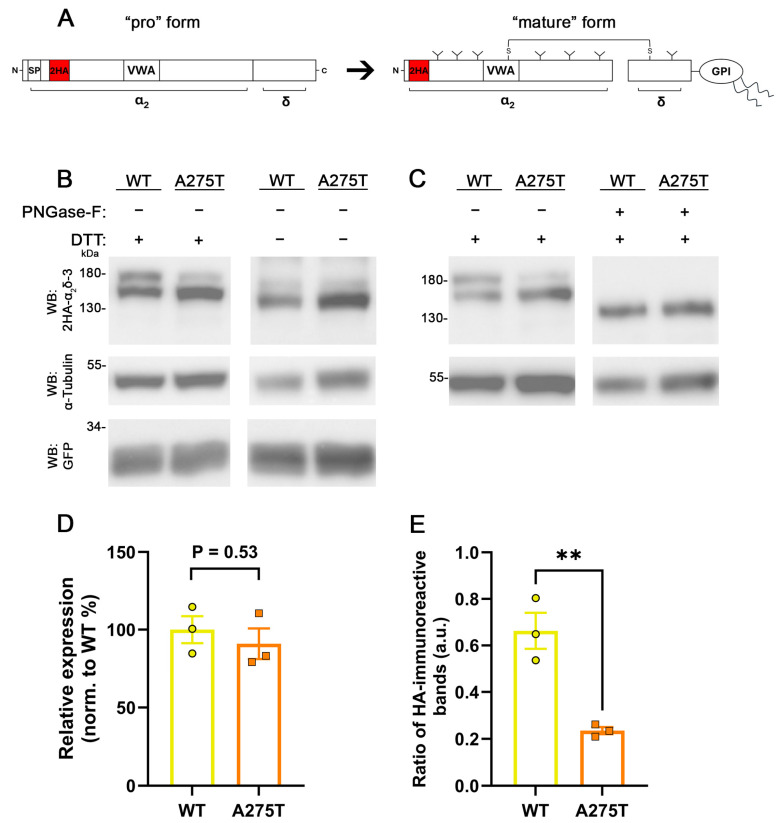
**The p.A275T mutation does not affect the overall expression levels of α_2_δ-3 but alters the glycosylation pattern.** (**A**) Schematic representation of “pro” form of α_2_δ (**left**) showing the approximate position of the signal peptide (SP), double HA tags (2HA), and VWA domain. Post-translational processing α_2_δ (mature form, **right**) includes glycosylation (Y), glycosyl-phosphatidyl inositol (GPI)-anchoring, proteolytic cleavage, and formation of multiple disulfide bonds between and within α_2_ and δ peptides. (**B**,**C**) Immunoblot of whole-cell lysates obtained from tsA201 cells transfected with 2HA-tagged WT or mutated α_2_δ-3 together with eGFP. α_2_δ-3 protein was detected with an anti-HA antibody (upper panel), anti-tubulin labelling was used as a loading control (middle panel), and anti-GFP for controlling transfection efficiency (lower panel). (**B**) Glycosylated and reduced α_2_δ-3 (lanes 1 and 2), glycosylated and unreduced α_2_δ-3 (lanes 3 and 4). (**C**) Glycosylated and reduced α_2_δ-3 (lanes 1 and 2), de-glycosylated and reduced α_2_δ-3 (lanes 3 and 4). Proteins were de-glycosylated with Peptide N-glycosidase (PNGase-F) and reduced with dithiothreitol (DTT). (**D**) Quantification of protein expression levels of α_2_δ-3 relative to GFP expression and normalized to WT expression. (**E**) The ratio of the intensities of the upper and lower anti-HA-immunoreactive bands under reducing conditions. Data were obtained from three independent transfections. Values of individual transfections (dots) and mean bars ± SEM (lines) are shown. **Statistics**: (**D**) unpaired two-tailed *t*-test, t_(4)_ = 0.69; *p* = 0.53; (**E**) unpaired two-tailed *t*-test, t_(4)_ = 5.4; *p* = 0.006 (**).

To confirm that the diminished neuronal membrane expression of α_2_δ-3_A275T does not result from a decreased protein expression, we analyzed total protein levels upon heterologous expression in tsA201 cells, similar to α_2_δ-1_R351T (confer Figure 4). The HA-epitope-tagged WT or mutated α_2_δ-3 were expressed together with soluble eGFP in tsA201 cells. Immunostaining of western blots (WB) against the HA-epitope revealed two distinct bands for WT α_2_δ-3 migrating at ~150 kDa and ~180 kDa (Figure 5B, lane 1). Anti-HA immunostaining of α_2_δ-3_A275T showed similar bands, however, there was an apparent shift in the relative intensity of the two bands, with the ~180 kDa band showing a lower and the ~150 kDa band showing a higher labelling intensity when compared with WT α_2_δ-3 (Figure 5B, lanes 1 and 2). Calculating the ratio of the upper relative to the lower HA-immunoreactive band demonstrates a significantly reduced ratio with mutated α_2_δ-3 (Figure 5E). However, analysis of the combined intensities of both bands shows a comparable average intensity of WT and mutated α_2_δ-3 immunolabeling (Figure 5D), implying that even though there is a change in the abundance of the respective protein variants, the total protein expression of α_2_δ-3_A275T is not affected.

The differences in the relative band intensities between WT and mutated α_2_δ-3 may suggest that the A275T mutation affects the post-translational modification of α_2_δ proteins, for example, the proteolytic processing. Unlike endogenous expression, heterologous expression of α_2_δ proteins has been shown previously to result in only partial cleavage into α_2_ and δ peptides [38] and, hence, the vast amount of α_2_δ proteins remains un-cleaved (confer Figure 5A). Because the reducing agent dithiothreitol (DTT) is a basic component of our routine WB protocol, the disulfide bonds between the α_2_ and δ peptides are expected to be resolved with DTT. Therefore, we hypothesized that the ~180 kDa band corresponds to un-cleaved α_2_δ-3, whereas the ~150 kDa band corresponds to the mature and cleaved α_2_ protein only (the HA-tag was cloned to the N-terminus right after the signal peptide [7]). Hence, our observation of the relative band intensities implies that the p.A275T mutation nudges α_2_δ-3 protein to undergo greater proteolytic cleavage than WT α_2_δ-3. To test this hypothesis, we omitted DTT to maintain the disulfide bonds intact and hence show only un-cleaved α_2_δ-3 and the mature disulfide-linked α_2_δ-3 protein. Contrary to our hypothesis, the non-reducing conditions resulted neither in a single HA-immunoreactive band (cleaved and un-cleaved α_2_δ-3) nor eliminated the apparent differences in the relative intensities of the upper and lower HA-immunoreactive bands of WT and mutated α_2_δ-3 (Figure 5B; lanes 3 and 4). However, in the non-reducing condition, both bands migrated at a lower protein size (~140 kDa and ~170 kDa) when compared to the reducing condition (~150 kDa and ~180 kDa, compare Figure 5B lanes 3 and 4 with lanes 1 and 2). This experiment demonstrates that the difference in the relative intensities of the upper and lower bands between WT and mutated α_2_δ-3 are not caused by differences in the proteolytic processing.

α_2_δ proteins are extensively post-translationally glycosylated [39], with 15 N-linked glycosylation sites identified in the cryo-EM structure of rabbit α_2_δ-1 [40]. Notably, two of these glycosylation sites have been shown to be crucial for the regulatory effects of α_2_δ subunits on calcium channels [41]. Hence, differences in the post-translational glycosylation pattern may provide an alternative explanation for the observed differences between WT and mutated α_2_δ-3. To further study the mechanism underlying the difference between mutated and WT α_2_δ-3 immunoreactive bands, we de-glycosylated the whole-cell lysates with PNGase-F prior to the SDS-PAGE. Following de-glycosylation, only one HA-immunoreactive band migrating at around ~135 kDa could be detected in both WT and mutated α_2_δ-3 (Figure 5C, lanes 3 and 4), demonstrating that the difference in the bands’ relative intensities was eliminated following de-glycosylation. With respect to the labelling pattern of DTT-treated samples (Figure 5B,C, lanes 1 and 2), this suggests that in tsA201 cells WT α_2_δ-3 exists in two distinct glycosylation patterns. Together, our data show that the A275T mutation affects the abundance of the two different glycosylation patterns and hence suggests that glycosylation is altered in mutated α_2_δ-3.

### 2.5. R351T and A275T Mutations in α_2_δ-1 and α_2_δ-3, Respectively, Do Not Compromise Current Properties of Ca_V_2.1 Channels

**Figure 6:**
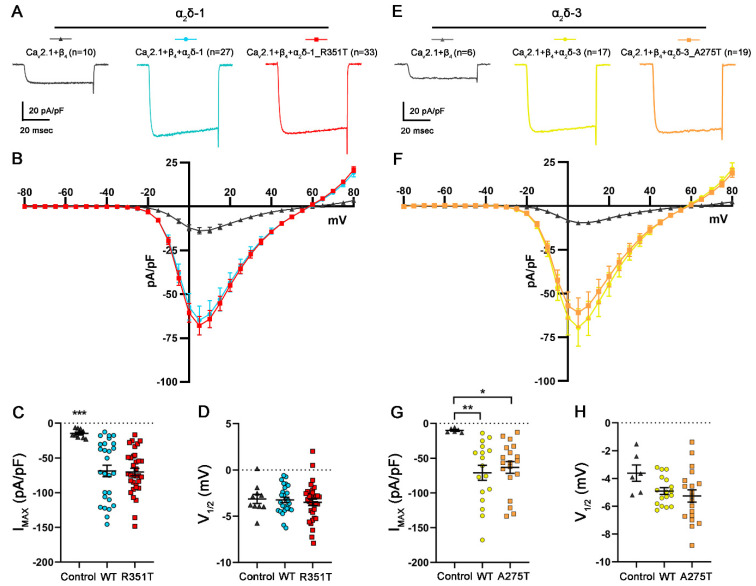
**Mutated α_2_δ proteins increase current densities of Ca_V_2.1 channels similar to WT α_2_δ.** (**A**–**D**) Calcium current properties of Ca_V_2.1 recorded from tsA201 cells co-transfected with Ca_V_2.1 and β_4_ alone as a control (control, gray triangles) or together with WT (WT, blue circles), or mutated α_2_δ-1 (R351T, red rectangles), and (**E**–**H**) from tsA201 cells transfected with Ca_V_2.1 and β_4_ alone as a control (control, gray triangles) or together with WT (WT, yellow circles), or mutated α_2_δ-3 (A275T, orange rectangles). 50 msec test pulses from a holding potential of −80 mV to +80 mV were applied in 5 mV increments. (**A**,**E**) Representative whole-cell Ca^2+^ current traces obtained at V_MAX_. Current–voltage relationships (**B**,**F**), peak current densities (**C**,**G**), and half-maximal activation potentials (**D**,**H**) are shown. **Statistics**: (**C**,**D**) One-way ANOVA with Tukey’s post hoc multiple comparison was performed on 10–33 recordings per condition obtained from three independent experiments. (**C**) Maximal current density, F_(2, 7)_ = 10.7; *p* < 0.0001, (**D**) half-maximal activation potential, F_(2, 67)_ = 0.25; *p* = 0.78. (**G**,**H**) ANOVA with Tukey’s post hoc multiple comparison was performed on 6–19 recordings per condition obtained from three independent experiments. (**G**) Maximal current density, F_(2,39)_ = 6.0; *p* = 0.006, (**H**) half-maximal activation potential, F_(2, 39)_ = 2.6; *p* = 0.09. Significances of post hoc tests between conditions are indicated in the graphs by asterisks (*** *p* < 0.001, ** *p* < 0.01, * *p* < 0.05).

α_2_δ proteins are traditionally considered as auxiliary subunits of VGCCs that enhance the expression of functional calcium channels at the plasma membrane and modulate biophysical channel properties (reviewed in [42,43]). It is widely established that heterologous co-expression of α_2_δ proteins increases the maximum current density of Ca_V_1 and Ca_V_2 channels. Because the single channel conductance was not changed by α_2_δ [44,45], the proposed mechanism underlying the α_2_δ subunit-induced increase in current density is an increase in forward trafficking coupled with a decrease in their turnover rate. The MIDAS motif within the VWA domain has been shown to play a key role in this mechanism [37]. Because the amino acids R351 and A275 are located in the VWA domain of α_2_δ, we hypothesized that p.R351T and p.A275T mutations may disrupt the channel-dependent function of α_2_δ. To test this hypothesis, we first studied the current properties of the P/Q-type channel Ca_V_2.1 by performing whole-cell patch clamp recordings in tsA201 cells co-transfected with Ca_V_2.1 and β_4_ without (control) or together with WT or mutated α_2_δ. As previously reported, both the co-expression of α_2_δ-1 or α_2_δ-3 lead to a ~5-fold increase in the current density compared to the omission of α_2_δ (control condition). More importantly, co-expression of mutated α_2_δ-1 and α_2_δ-3 proteins resulted in similarly increased current densities (Figure 6 and Table 2). These results show that despite the strong reduction in their membrane expression (confer Figure 2 and Figure 4), α_2_δ-1_R351T and α_2_δ-3_A275T can interact with Ca_V_2.1 channels and enhance their functional membrane expression.

### 2.6. R351T and A275T Mutations in α_2_δ-1 and α_2_δ-3, Respectively, Do Not Compromise the Current Properties of L-Type Calcium Channels

**Figure 7:**
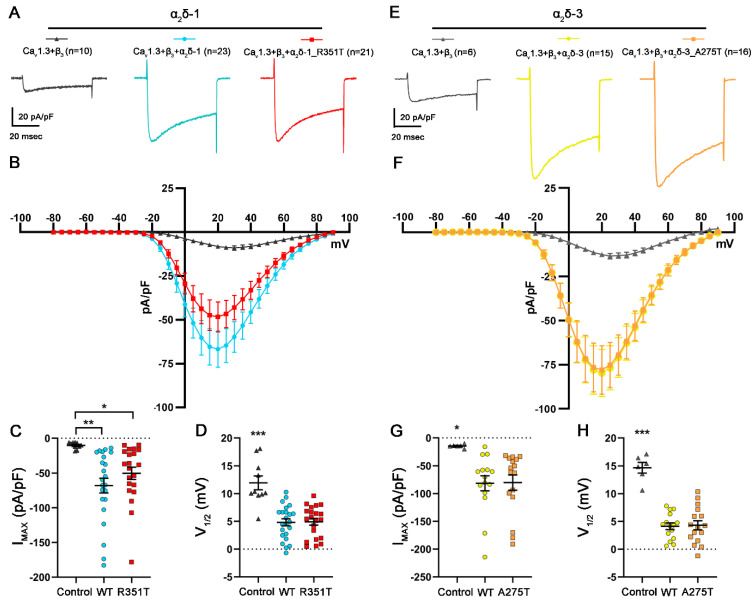
**Mutated α_2_δ proteins modulate current properties of Ca_V_1.3 channels similar to WT α_2_δ.** (**A**–**D**) Calcium current properties of Ca_V_1.3 channels recorded from tsA201 cells transfected with Ca_V_1.3 and β_3_ alone as control (control, gray triangles) or together with WT (WT, blue circles), or mutated α_2_δ-1 (R351T, red rectangles), and (**E**–**H**) recordings from tsA201 cells transfected with Ca_V_1.3 and β_3_ alone as control (control, gray triangles) or together with WT (WT, yellow circles), or mutated α_2_δ-3 (A275T, orange rectangles). 50 msec test pulses from a holding potential of −80 mV to +90 mV were applied in 5 mV increments. (**A**,**E**) Representative whole-cell Ca^2+^ current traces obtained at V_MAX_. Current–voltage relationships (**B**,**F**), peak current densities (**C**,**G**), and half-maximal activation potentials (**D**,**H**) are shown. **Statistics**: (**C**,**D**) One-way ANOVA with Tukey’s post hoc multiple comparison was performed on 10–23 recordings per condition obtained from three independent experiments. (**C**) Maximal current density, F_(2, 51)_ = 6.6; *p* = 0.0028, (**D**) half-maximal activation potential, F_(2, 51)_ =20.6; *p* < 0.0001. (**G**,**H**) ANOVA with Tukey’s post hoc multiple comparison was performed on 6–16 recordings per condition obtained from two independent experiments. (**G**) Maximal current density, F_(2, 34)_= 4.5; *p* = 0.019, (**H**) half-maximal activation potential, F_(2, 34)_ =34.9; *p* < 0.0001. Significances of post hoc tests between conditions are indicated in the graphs by asterisks (*** *p* < 0.001, ** *p* < 0.01, * *p* < 0.05).

In addition to increasing the functional membrane expression, α_2_δ subunits can further modulate the voltage dependence and kinetics of L-type calcium currents (reviewed in [42,46]). Whereas the main modulatory effect of α_2_δ proteins on P/Q-type channels is through increasing the functional membrane expression of the channels, on L-type channels, they modulate the voltage dependence and current kinetics with smaller effects on the membrane incorporation [47,48,49]. The size of these two effects depends on the different combinations of α_1_ and α_2_δ isoforms co-expressed. Although it is still unclear how the α_2_δ proteins confer these effects on the channels, it may relate to subtle isoform-specific differences in α_1_/α_2_δ interactions [13]. Therefore, we next tested if p.R351T and p.A275T mutations affect the current properties of L-type Ca_V_1.3 channels. Consistent with previous studies, co-expression of WT α_2_δ left-shifted the voltage-dependence of activation and increased current amplitudes of Ca_V_1.3 (Figure 7 and Table 3, refer to WT). Co-expression of mutated α_2_δ-1 and α_2_δ-3 resulted in similar increases in current densities and similar left-shifts of voltage-dependences of activation of Ca_V_1.3 (Figure 7 and Table 3, refer to R351T and A275T).

**Figure 8:**
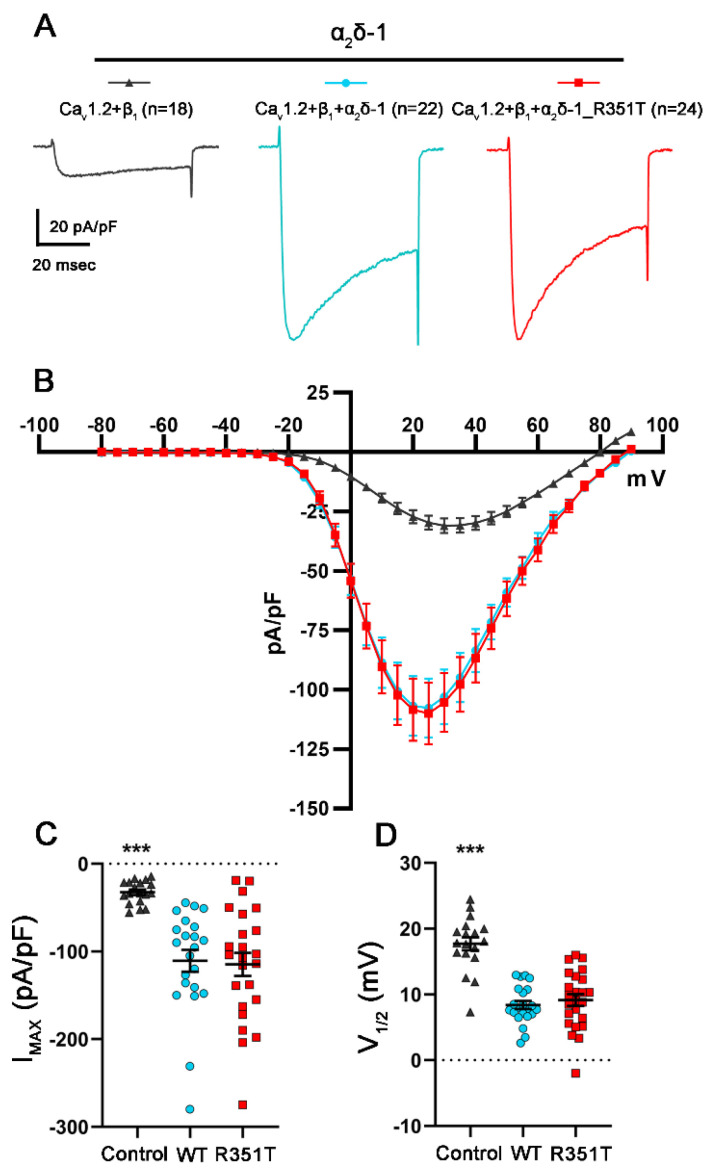
**α_2_δ-1_R351T modulates current properties of Ca_V_1.2 channels like WT α_2_δ-1.** (**A**–**D**) Calcium current properties of Ca_V_1.2 channels recorded from tsA201 cells transfected with Ca_V_1.2 and β_1_ alone as control (control, gray triangles) or together with WT (WT, blue circles), or mutated α_2_δ-1 (R351T, red rectangles). 50 msec test pulses from a holding potential of −80 mV to +90 mV were applied in 5 mV increments. (**A**) Representative whole-cell Ca^2+^ current traces obtained at V_MAX_. Current–voltage relationships (**B**), peak current densities (**C**), and half-maximal activation potentials (**D**) are shown. **Statistics**: One-way ANOVA with Tukey’s post hoc multiple comparison was performed on 18–24 recordings per condition obtained from three independent experiments. (**C**) Maximal current density, F_(2, 61)_ = 14.9; *p* < 0.0001, (**D**) half-maximal activation potential, F_(2, 61)_ =34.7; *p* < 0.0001. Significances of post hoc tests between conditions are indicated in the graphs by asterisks (*** *p* < 0.001).

Structural interface analysis between different α_1_ and α_2_δ isoforms suggests preferences in complex formation [13]. Moreover, single molecule tracking analysis revealed differences in the engagement of α_2_δ proteins with α_1_ subunits [50]. These studies suggest distinct, albeit subtle, differences in the interaction of α_2_δ isoforms with different Ca_V_ α_1_ subunits. Hence, we further investigated the potential consequences of the p.R351T mutation on the Ca_V_1.2 channel, another neuronal L-type channel subtype. Like the observation with the L-type channel Ca_V_1.3, and indistinguishable from WT α_2_δ-1, co-expression of mutated α_2_δ-1 resulted in ~3.5-fold increase in the maximal current density and left-shifted the voltage-dependence of activation to more negative potentials, when compared to the control condition without α_2_δ-1 (Figure 8 and Table 4, refer to WT and R351T). Taken together, the mutated α_2_δ proteins showed unaltered channel-dependent modulatory functions.

### 2.7. Reduced Presynaptic Localization of α_2_δ-1_R351T and α_2_δ-3_A275T but Unaltered Trans-Synaptic Signaling

**Figure 9:**
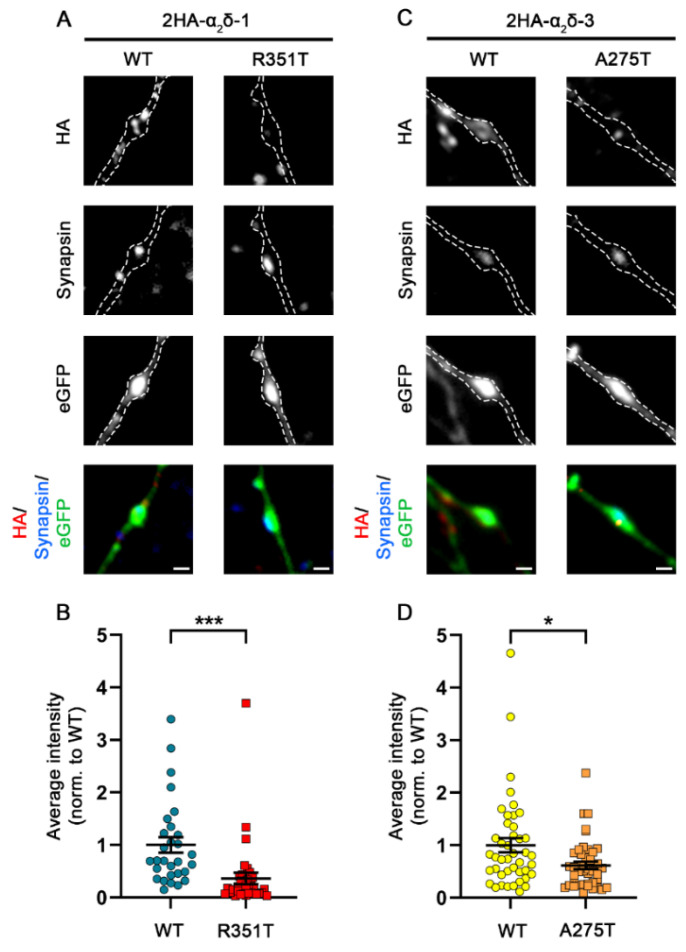
**Strongly reduced presynaptic targeting of α_2_δ-1_R351T and α_2_δ-3_A275T.** (**A**,**C**) Representative presynaptic boutons of cultured hippocampal neurons transfected with eGFP together with WT or mutated α_2_δ. Presynaptic boutons are identified by the clustering of synapsin proteins (blue) within axonal varicosities as visualized by the eGFP fluorescence (outlined by a dashed line). Live-cell staining reveals that mutated α_2_δ-1 and α_2_δ-3 proteins show a lower expression at the surface of presynaptic boutons compared to WT α_2_δ proteins (red). (**B**,**D**) Average fluorescence intensity measurements of the HA signal in transfected boutons revealed a strong reduction in presynaptic membrane expression of mutated α_2_δ compared to WT α_2_δ proteins. Graph shows mean values of minimum five synapses of individual cells (dots) and means ± SEM (lines). Data were obtained from at least three independent culture preparations. **Statistics**: (**B**) 29 and 35 cells expressing WT or mutated HA-tagged α_2_δ-1 were analyzed, respectively. Unpaired *t*-test, t_(62)_ = 3.5; *** *p* = 0.0008. (**D**) 43 and 44 cells expressing WT or mutated HA-tagged α_2_δ-3 were analyzed, respectively. Unpaired *t*-test, t_(85)_ = 2.6; * *p* = 0.01. Scale bars, 1 µm.

The autism-associated mutations in α_2_δ-1 and α_2_δ-3 strongly affect neuronal and heterologous membrane expression (confer Figure 2 and Figure 4), but they do not compromise membrane trafficking and function of presynaptic Ca_V_2.1 channels upon heterologous co-expression (confer Figure 6). However, presynaptic α_2_δ-1 and α_2_δ-3 isoforms have been shown to play a pivotal role in synapse formation and differentiation [8]. Thus, presynaptic and/or channel-independent functions of α_2_δ proteins may depend stronger on the local expression density than the effects observed upon heterologous expression. Therefore, we next studied the consequences of p.R531T and p.A275T mutations on the presynaptic localization of α_2_δ proteins. Quantitative analysis of presynaptic (synapsin positive) boutons expressing HA tagged α_2_δ proteins showed a reduced surface expression of α_2_δ-1_R351T and α_2_δ-3_A275T compared to WT α_2_δ-1 and α_2_δ-3, respectively (Figure 9B,D), which is in line with the overall reduction in neuronal membrane expression.

**Figure 10:**
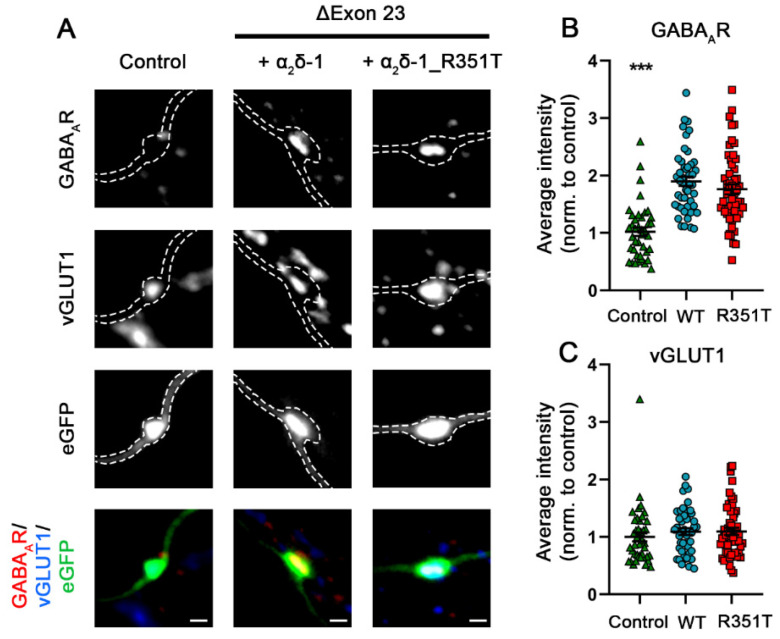
**Unaltered trans-synaptic coupling of α_2_δ-1_R351T_ΔE23 to postsynaptic GABA_A_R.** Synapses in hippocampal neurons transfected with soluble eGFP as control (green triangles), or together with either WT (blue circles) or mutated (red rectangles) α_2_δ-1_ΔE23, were identified by immunofluorescent labelling of presynaptic vGLUT1 and postsynaptic GABA_A_-receptors. (**A**) Both overexpression of WT and mutated α_2_δ-1_ΔE23 leads to the formation of mismatched synapses as detected by postsynaptic GABA_A_ receptor clusters opposite vGLUT1 positive glutamatergic terminals (A, α_2_δ-1, α_2_δ-1_R351T). Quantifications of immunofluorescence intensities of GABA_A_ receptor (**B**) and vGLUT1 (**C**) labelling show values for individual cells (dots) and means ± SEM (lines). Cells were obtained from three independent culture preparations. Values were normalized to the fluorescent intensities of the control condition within each culture preparation. **Statistics**: ANOVA with Tukey’s post hoc multiple comparison was performed on 41–52 cells per condition. GABA_A_R: F_(2, 136)_ = 29.5; *p* < 0.0001, vGLUT1: F_(2, 136)_ = 0.4; *p* = 0.67. Significances of post hoc tests between the control and the α_2_δ-1_ΔE23 conditions are indicated in the graphs by asterisks (*** *p* < 0.001). Scale bars, 1 µm.

Presynaptic overexpression of an α_2_δ-2 splice variant lacking exon 23 (α_2_δ-2_ΔE23) in GABAergic and glutamatergic synapses leads to a strongly increased clustering of postsynaptic GABA_A_Rs [7]. In glutamatergic synapses this induces aberrant axonal wiring of glutamatergic presynaptic boutons with GABAergic postsynaptic sites, resulting in mismatched synapses [7]. Similar to α_2_δ-2, alternative splicing of exon 23 is also observed in α_2_δ-1 but not in α_2_δ-3. Therefore, we tested whether presynaptic expression of α_2_δ-1_R351T in hippocampal neurons alters trans-synaptic signaling. To this end, primary cultured hippocampal neurons were transfected with eGFP alone (control) or together with either WT or mutated α_2_δ-1, and subsequently stained for marker proteins of glutamatergic presynaptic (vGLUT1) and GABAergic postsynaptic (GABA_A_Rs) compartments. In a normal synapse, vGLUT1 positive presynaptic boutons do not co-localize with postsynaptic GABA_A_Rs (Figure 10A, left panel, Control). In stark contrast, presynaptic glutamatergic boutons, identified by vGLUT1 clustering, are co-localizing with marked clusters of postsynaptic GABA_A_Rs in neurons overexpressing α_2_δ-1_ΔE23 (Figure 10A, middle panel). Hence, presynaptic expression of α_2_δ-1_ΔE23 induces aberrant trans-synaptic signaling like it was previously shown for α_2_δ-2_ΔE23 [7]. Despite the strong reduction in presynaptic localization of mutated α_2_δ-1, neurons transfected with α_2_δ-1_ΔE23_R351T were able to recruit GABA_A_Rs opposite glutamatergic nerve terminals to the same levels as neurons transfected with WT α_2_δ-1 (Figure 10A, right panel and Figure 10B, quantification). Together, this shows that the p.R351T mutation does not compromise the trans-synaptic signaling of α_2_δ-1 and suggests that even very small amounts of α_2_δ-1 expressed on the membrane of presynaptic terminals are sufficient to mediate this trans-synaptic function.

## 3. Discussion

ASDs cover a wide range of disease expressions and severities [1] and are largely defined as synaptopathies [51]; however, the detailed disease mechanisms are still elusive. α_2_δ proteins are subunits of VGCCs and are critical and redundant regulators of glutamatergic synapse formation [8] and trans-synaptic signaling [7,52]. Hence, to shed light on the cellular disease mechanisms, we here analyzed the consequences of autism-associated mutations in α_2_δ-1 and α_2_δ-3 on calcium channels and trans-synaptic functions. The experiments demonstrate a strongly reduced membrane expression and presynaptic targeting of the mutated α_2_δ proteins without affecting total protein expression levels. Importantly, this reduced membrane expression did not result in altered functions of postsynaptic L-type (Ca_V_1.3 and Ca_V_1.2) and presynaptic P/Q-type (Ca_V_2.1) channels upon heterologous co-expression. Surprisingly, the p.R351T mutation neither compromises the trans-synaptic coupling of presynaptic α_2_δ-1 with postsynaptic GABA_A_R, a function that is proposed to rely on extracellular protein–protein interactions. Finally, western blot analysis indicates that the p.A275T mutation changes the glycosylation pattern of α_2_δ-3. Together, our data suggest that the pathophysiological mechanism may not be mediated primarily via defective calcium or trans-synaptic signaling. It may, however, involve subtle changes in synaptic connectivity that need to be addressed in future experiments employing a conditional knockout background.

### 3.1. The Autism-Associated Mutations in α_2_δ-1 and α_2_δ-3 Reduce Protein Surface Expression but Do Not Compromise the Current Properties of L- and P/Q-Type Calcium Channels

Given the critical position of the two substitutions (Figure 1), it is not surprising that the mutated proteins show a decrease in membrane expression compared to the corresponding WT proteins (Figure 2). This observation might be the result of reduced forward trafficking from the endoplasmic reticulum (ER) or an increased internalization rate from the plasma membrane. To date, a wealth of heterologous co-expression studies showed that α_2_δ subunits share a canonical role in enhancing the functional membrane expression of Ca_V_1 and Ca_V_2 channels. In addition, α_2_δ proteins differentially modulate current properties of distinct α_1_ subunits [43,49,53]. A structure–function study showed that the MIDAS motif within the VWA domain of α_2_δ is crucial for augmenting the cell surface density of Ca_V_ channels [37]. Moreover, the modulatory effects of α_2_δ proteins on the voltage-dependent properties of Ca_V_ channels are likely mediated by increasing the voltage sensitivity of the voltage-sensing domains (VSDs) I–III [54]. These studies were further underpinned by the cryo-EM structure of the Ca_V_1.1 channel complex, showing that the binding interface between α_2_δ and α_1_ subunits involves the VWA and cache1 domains of α_2_δ-1, and the extracellular loops of the repeats I–III of α_1_. However, the p.R351T and p.A275T mutations did not compromise the functional membrane expression of Ca_V_ channels (Figure 6, Figure 7 and Figure 8; Table 2, Table 3 and Table 4), despite their pivotal location within the VWA domain and proximity to the MIDAS motif. This suggests that a normal or stable membrane expression of α_2_δ proteins is not a prerequisite for mediating channel membrane trafficking and that these processes may be independently regulated [49]. This hypothesis is supported by evidence that α_2_δ subunits enhance the membrane expression of the channel through an interaction occurring in the ER rather than at the plasma membrane [37,55]. Notably, the mutated α_2_δ proteins were able to modulate the voltage-dependence of activation of L-type channels like WT α_2_δ proteins (Figure 7 and Figure 8; Table 3 and Table 4; ~6–9 mV shift), indicating that a stable α_1_–α_2_δ interaction is not a prerequisite for channel modulation. Along these lines, a single molecule tracking study suggests a weak and transient association between α_1_ and α_2_δ, with the latter showing a significantly higher surface mobility [50]. However, whether these mutations modulate calcium channels also in their native environment, e.g., in pre- or postsynaptic compartments, needs to be further explored in homologous expression systems, preferentially by reconstituting specific α_2_δ knockout neurons.

### 3.2. The R351T Mutation in α_2_δ-1 Does Not Compromise Trans-Synaptic Signaling

The cryo-EM structures of different VGCC complexes [40,56,57,58,59] emphasize the large extracellular part of α_2_δ proteins that provide numerous potential interaction sites with extracellularly exposed synaptic and/or matrix proteins. Indeed, both pre- and postsynaptic α_2_δ proteins have been shown to be critical for glutamatergic synapse formation and differentiation [37,51], and presynaptic α_2_δ proteins potently regulate the abundance of postsynaptic GABA_A_Rs by a trans-synaptic mechanism [7,13,60]. Taken together, these studies support the existence of functional roles for α_2_δ independent from the calcium channel complex. However, in our present study, the drastic reduction in presynaptic localization of α_2_δ-1_R351T did not alter trans-synaptic coupling with postsynaptic GABA_A_R (Figure 10). While this finding does not contribute to our understanding of the pathophysiological role of α_2_δ-1_R351T, it provides insights into the physiological mechanism of this trans-synaptic signaling. The fact that the extent of presynaptic membrane expression of WT or mutant α_2_δ-1 does not correlate with postsynaptic GABA_A_R recruitment indicates that the trans-synaptic interaction between α_2_δ proteins and GABA_A_Rs may require an additional mediator, e.g., an indirect signaling molecule [7]. Alternatively, this observation may imply that a transient expression of α_2_δ on the cell surface during early developmental stages is enough to recruit post-synaptic GABA_A_Rs [60].

### 3.3. The A275T Mutation Alters the Glycosylation Pattern of α_2_δ-3

Both α_2_ and δ peptides are heavily glycosylated (15 N-linked glycosylation sites as predicted by the cryo-EM structure of rabbit α_2_δ-1 [40]), and at least two of these glycosylation sites were found to be critical for the α_2_δ subunit mediated regulation of Ca_V_ channels [41]. Additionally, the initial biochemical investigation of the α_2_δ-1 subunit revealed size heterogeneity attributed to variable N-linked glycosylation [39], suggesting that α_2_δ proteins may exist in multiple glycosylation states. Our study indicates that the p.A275T mutation induces changes in the glycosylation pattern of α_2_δ-3 (Figure 5). These potential changes, however, did not affect the regulation of heterologously expressed calcium channels (Figure 6 and Figure 7). The amino acid alanine at position 275 is not a potential glycosylation site, neither in the mouse (UniProt identifier: Q9Z1L5) nor in the human α_2_δ-3 protein (UniProt identifier: Q8IZS8). Moreover, most computational tools designed for predicting protein glycosylation based on amino acid sequences did not suggest any changes in the glycosylation patterns of the α_2_δ-3_A275T mutant when compared to the WT protein (GlycoPP, [61]; NetNGlyc-1.0, [62]). All these prediction tools rely only on consensus sequences for N- or O-glycosylation; however, the p.A275T mutation may lead to changes in protein folding, rendering certain, yet unidentified, amino acids unavailable for glycosidic binding. In light of the potential pathogenicity of the p.A275T mutation, future studies are needed to analyze the prevalence of the altered glycosylation pattern in a native environment (e.g., by reconstitution of conditional knock-out mice or knock-in mice carrying the p.A275T mutation), to identify the specific sites involved in this glycosylation pattern (e.g., by mass spectrometry [63]), and to ultimately assess the functional consequence of the altered glycosylation.

### 3.4. Potential Disease Mechanisms Underlying α_2_δ Protein-Associated Autism Spectrum Disorders

Autism spectrum disorders constitute a “synaptopathy” [51]. In light of the recently identified roles of α_2_δ proteins in synapse formation [8,13], synaptic connectivity [7,52], and dendritic spine maturation [14], synaptic dysfunctions caused by α_2_δ mutations provide a theoretical pathophysiological link to ASD. Therefore, synaptic dysfunction could be caused by altered pre- or postsynaptic calcium channel regulation or by defects in synapse development, wiring, and differentiation. α_2_δ-1 and α_2_δ-3 can differentially modulate excitatory and inhibitory connectivity during the development of hippocampal neurons [12]. A reduced neuronal surface expression of α_2_δ-1 and α_2_δ-3 caused by the R351T and A275T mutations during critical developmental periods could impact the excitatory/inhibitory balance, a condition that is notably associated with ASDs [64,65]. However, our present study does not provide a proof for the pathogenicity of the studied mutations as they affected neither calcium channel modulation nor trans-synaptic signaling, in contrast to an epilepsy-linked mutation in α_2_δ-2 [60]. Alternatively, the mutations may only cause subtle changes in synaptic connectivity or affect specific synaptic connections, which cannot be reproduced in a model of dispersed cultured hippocampal neurons. Elucidating such subtle changes is complicated by the facts that presynaptic α_2_δ isoforms are highly redundant in their role as synaptic organizers [8,13] and that the majority of brain regions simultaneously express α_2_δ-1, α_2_δ-2, and α_2_δ-3 [11,66]. A previous study provided one potential pathophysiological explanation [14], which suggested that the p.R351T mutation hinders cell surface trafficking of α_2_δ-1, a finding we could validate and quantify. This study suggested that the reduced surface expression of α_2_δ-1 may limit the accessibility of potential extracellular ligands, thereby modulating synapse formation and dendritic spine maturation.

The mutations p.R351T in α_2_δ-1 and p.A275T in α_2_δ-3 have previously been identified in human patients and predicted to be pathogenic. Autism presents with a broad spectrum of disorders ranging from subtle behavioral modifications to severe presentations that strongly affect cognition and social interactions [67]. Current animal models of autism, however, are based on human mutations linked to very severe disease presentations, often associated with complex neurological and neurodevelopmental disorders. Hence, among other comorbidities, these patients are also diagnosed with ASD (e.g., FMR1; reviewed in [68]). This highlights the need to establish experimental tools to study subtle synaptic dysfunctions in the native environment of a mammalian brain, as such modifications could explain the pathophysiology underlying α_2_δ protein associations with ASD and, hence, future treatment paradigms. These studies may involve the generation of novel conditional knock-out or knock-in mouse models, allowing modifications of specific brain regions, which will require extensive behavioral and structural characterization.

## 4. Materials and Methods

### 4.1. Animals

Animal breeding of wildtype BALB/c mice was performed at the Medical University Innsbruck in compliance with EU and national regulations and was approved by the Austrian Federal Ministry of Science, Research and Economy in accordance with §16 TVG 2012, license numbers BMWF-66.011/0017-II/3b/2014 and BMWF-66.011/0067-II/3b/2014. Mice were maintained at the central animal facility in Innsbruck in groups of 3–5 per cage under standard housing conditions, with food and water available ad libitum on a 12 h light/dark cycle. Mice used for breeding were 2 to 14 months old. According to the RRR principle, the number of mice used was kept to the minimum necessary for a statistical representative analysis, which was comparable to the numbers reported in previous studies.

### 4.2. Expression Vectors and Cloning Procedures

All plasmids used to transfect primary cultured hippocampal neurons were cloned into a eukaryotic expression plasmid containing a neuronal chicken β-actin promoter (pβA) to improve neuronal expression. Newly generated constructs were verified by Sanger sequencing (Eurofins Genomics). The cloning procedures to generate the following plasmids were described previously: pβA-eGFP [69], pβA-α_2_δ-1-v1 [13], pβA-α_2_δ-3 and pβA-2HA-α_2_δ-3 [8], pGFP-Ca_V_1.3 [70], pCMV-Ca_V_2.1 [71], β_3_ [72], β_4e_ [73], and pβA-Ca_V_1.2 [74].

**pβA-α_2_δ-1-v1_R351T:** The p.R351T mutation was introduced with 19 bp overlapping mutagenesis primers in two separate PCR reactions, using pβA-α_2_δ-1-v1 as a template. For the first PCR reaction, the forward mutagenic primer sequence was 5′-aatgtttccacagctaattgcaataagatt-3′, and the reverse flanking primer sequence was 5′-tgcttgggtcaatctctcca-3′. For the second PCR reaction, the forward flanking primer sequence was 5′-tggccttgactctgacactt-3′, and the reverse mutagenic primer sequence was 5′-gcaattagctgtggaaacattataattaag-3′. The two separate PCR products were then used as templates for a final PCR reaction with the two flanking primers to connect the nucleotide sequences. The resulting fragment was then AflII/BglII digested and ligated into the corresponding site of pβA-α_2_δ-1-v1 yielding pβA-α_2_δ-1-v1_R351T.

**pβA-2HA-α_2_δ-1-v1:** pβA-α_2_δ-1-v1 was BglII/SalI digested, and the fragment containing the coding sequence of α_2_δ-1-v1 was ligated into the corresponding site of pβA-2HA-α_2_δ-1-v2 (mouse α_2_δ-1-v2 cDNA sequence with a double hemagglutinin (2HA) tag at the N-terminus after the predicted signal peptide cleavage site, [7]) yielding pβA-2HA-α_2_δ-1-v1.

**pβA-2HA-α_2_δ-1-v1_R351T:** pβA-α_2_δ-1-v1_R351T was BglII/SalI digested, and the fragment containing the p.R351T mutation was ligated into the corresponding site of pβA-2HA-α_2_δ-1-v1 yielding pβA-2HA-α_2_δ-1-v1_R351T.

**pβA-α_2_δ-3_A275T:** The p.A275T mutation was introduced with 21 bp overlapping mutagenesis primers in two separate PCR reactions, using pβA-α_2_δ-3 as a template. For the first PCR reaction, the forward mutagenic primer sequence was 5′-gcttgaccatcaccaagcaaacagtgtcctcaatac-3′, and the reverse flanking primer sequence was 5′-acacccagaagaatgccctt-3′. For the second PCR reaction, the forward flanking primer sequence was 5′-gttcggcttctggcgtgt-3′, and the reverse mutagenic primer sequence was 5′-tgtttgcttggtgatggtcaagcggagtcctttcat-3′. The two separate PCR products were then used as templates for a final PCR reaction with the two flanking primers to connect the nucleotide sequences. The resulting fragment was then BglII/NotI digested and ligated into the corresponding site of pβA-α_2_δ-3 yielding pβA-α_2_δ-3_A275T.

**pβA-2HA-α_2_δ-3_A275T:** pβA-α_2_δ-3_A275T was BsrGI/XhoI digested, and the fragment containing the p.A275T mutation was ligated into the corresponding site of pβA-2HA-α_2_δ-3 yielding pβA-2HA-α_2_δ-3_A275T.

**pβA-β_1b_-eGFP:** to generate the C-terminally eGFP-tagged construct, β_1b_-eGFP, the cDNA coding for rat β_1b_ (Genbank X61394) was isolated from pβA-β_1b_-V5 [75] by HindIII/BglII digest, and inserted in the corresponding sites of pβA-β_2a_-eGFP [76].

### 4.3. Primary Culture and Transfection of Hippocampal Neurons

Low-density hippocampal cultures were generated from embryonic BALB/c mice aged between 16.5 and 18 days, as described previously [7,69,77]. Briefly, 2–8 hippocampi were dissected in cold Hank’s balanced salt solution (HBSS, Gibco, Grand Island, New York) and dissociated by 2.5% trypsin (Gibco) treatment and subsequent trituration. Dissociated neurons were plated at a density of ~3500 cells/cm^2^ on five 18 mm glass coverslips (Marienfeld Superior, Lauda-Königshofen, Germany) coated with poly-l-lysine (Sigma-Aldrich, St. Louis, MO, USA). After the attachment of neurons for 3–4 h, coverslips were transferred neuron-side down into a 60 mm culture dish containing a feeder monolayer of glia. Glial proliferation was inhibited with 5 µM Ara-C (Sigma-Aldrich, St. Louis, MO, USA) 3 days post-plating. Neurons and glia were maintained in NBKO (serum-free neurobasal medium (Gibco) supplemented with Glutamax (Gibco) and B-27 (Gibco)) that was changed weekly by replacing 1/3 of the volume with fresh maintenance medium. On DIV 6, neurons were transfected using Lipofectamine 2000 (Thermo Fisher Scientific, Waltham, MA, USA) as described previously [69]. A total of 1.5 µg of DNA was used for co-transfections at equimolar ratios. Neurons were processed for immunolabelling experiments between DIV 21 and 25.

### 4.4. Cell Culture and Transfection of tsA201 Cells

Human embryonic kidney (HEK)-293 subclone stably expressing SV40 temperature-sensitive T antigen (tsA201) cells (ECACC cat. No. 96121229, RRID: CVCL_2737), were cultured in Dulbecco’s modified Eagle’s medium (DMEM; Gibco), completed with 10% FBS (Gibco), 0.1 U/mL penicillin, and 0.1 μg/mL streptomycin (PenStrep, Gibco), and were maintained at 37 °C in a humidified incubator with 5% CO_2_. Cells were split when they reached ~80% of confluence using 0.5% trypsin-EDTA (Gibco), and the cell’s passage number did not exceed 20 passages. For whole-cell patch-clamp recordings, tsA201 cells were transiently transfected with α_1_ and β subunits as a control condition or together with WT or mutated α_2_δ subunits at equimolar ratios using FuGeneHD transfection reagent (Promega, Fitchburg, WI, USA), according to the manufacturer protocol. When the β subunit was not fused to GFP, eGFP was co-transfected to identify positively transfected cells. One day after transfection, cells were detached and replated at very low density on poly-l-lysine-coated 35 mm Petri dishes and kept at 30 °C and 5% CO_2_. Cells were used for electrophysiology experiments 48–72 h after transfection. For western blot experiments, cells were plated on 60 mm Petri dishes and transfected with 1 µg 2HA-tagged α_2_δ together with 0.5 µg eGFP. Experiments were performed 48 h after transfection.

### 4.5. Immunocytochemistry and High-Resolution Fluorescence Microscopy

The permeabilized and live-cell immunolabeling of neurons was performed as described previously [7,8,69,74]; information on primary and secondary antibodies is summarized in Table 5. For permeabilized staining, neurons were fixed with 4% paraformaldehyde and 4% sucrose in PBS (pF) for 20 min at room temperature, washed, and incubated for 30 min in 5% normal goat serum in PBS, containing 0.2% bovine serum albumin (BSA) and 0.2% Triton X-100 (PBS/BSA/Triton), to enable membrane permeabilization. Primary antibodies diluted in PBS/BSA/Triton were applied overnight at 4 °C and detected by fluorochrome-conjugated Alexa secondary antibodies incubated for 1 h at room temperature. For live-cell surface staining of HA-tagged α_2_δ proteins, transfected neurons were incubated with rat anti-HA antibodies diluted in the glia-conditioned neurobasal medium for 10 min at 37 °C, following quick rinsing in warm HBSS and fixation with pF for 10 min at room temperature. Subsequent washing and blocking steps, and 1 h incubation with fluorochrome-conjugated secondary goat anti-rat Alexa Fluor 594 antibodies, were conducted with PBS and PBS/BSA, respectively. After this first round of labeling, cells were fixed in pF for 5 min, then permeabilized by half an hour incubation in blocking solution containing 5% normal goat serum in PBS/BSA/Triton and subsequently incubated with primary mouse anti-synapsin antibodies overnight at 4 °C and detected with goat anti-mouse Alexa Fluor 350 antibodies. Coverslips were mounted on microscopy slides neuron-side down in DABCO glycerol solution (Carl Roth, Karlsruhe, Germany). tsA201 cells plated on PLL-treated coverslips and transfected with HA-tagged α_2_δ proteins were similarly immunolabeled to quantify the membrane expression of α_2_δ proteins (excluding the subsequent permeabilized staining for synapsin). Cells were imaged with a BX53 microscope (Olympus, Tokio, Japan) using a 60× 1.42 numerical aperture (NA) oil-immersion objective lens. Then, 14-bit grayscale images were recorded with a cooled CCD camera (XM10; Olympus) using cellSens Dimension software (Olympus) and further analyzed in MetaMorph software (Molecular Devices, Sunnyvale, CA, USA) or ImageJ software (National Institute of Health). Images of randomly selected well differentiated and positively transfected neurons were acquired with the same exposure and zero gain settings for all conditions within an experiment. Only cells with medium-level eGFP expression were selected for analysis, and strongly overexpressing cells (based on eGFP levels) were excluded from the analysis. Figures were assembled in Adobe Photoshop CS6 using linear adjustments to correct black level and contrast.

### 4.6. Image Analysis and Quantification

To analyze the effects of homologous presynaptic expression of WT or mutated α_2_δ-1 subunit on synapse composition of cultured hippocampal neurons, images from triple-fluorescence labelling were acquired from the eGFP (green), GABA_A_R_β2/3_ (red), and vGLUT1 (blue) channels. Images were analyzed with a custom programmed and semi-automated MetaMorph journal (Molecular Devices), as described previously [7,8]. For the individual synapses in each channel, the following parameters were detected in a blinded manner: eGFP threshold area as a measure for bouton size and average and integrated fluorescence intensities, providing information on the size and intensity of clusters. A modified protocol was employed to assess the presynaptic targeting of HA-tagged α_2_δ, as its staining pattern did not directly co-localize with the presynaptic eGFP signal [8]. To mitigate potential false positives or negatives, the presynaptic region of interest (ROI) was expanded by 0.5 μm. For each neuron, an average fluorescence value from a minimum of 10 presynaptic varicosities was calculated and plotted in GraphPad Prism 8. The fluorescent intensity of live-cell stained HA-tagged α_2_δ in the three main compartments of neurons was determined by measuring the fluorescent intensity along an outlined axon, dendrite, and cell soma. For each condition a minimum of 30 cells were considered from at least three independent culture preparations. All values were additionally normalized to the control or WT condition. Similarly, the surface expression of HA-tagged α_2_δ in tsA201 cells was quantified by measuring the average intensity of HA signals in isolated cells. To quantify the fluorescent intensity of immunostained HA-tagged α_2_δ in permeabilized neurons, the mean fluorescent intensity of the soma, dendrites, and axons was measured. To this end, the anti-HA images were thresholded to include soma, dendrites, and axons. Subsequently the thresholded region was converted into an ROI, and a background ROI outside the neuron was defined. Both ROIs were transferred to the original anti-HA and eGFP images and the average background-subtracted intensities of the ROIs were measured for each individual neuron.

### 4.7. Western Blotting

To determine total protein levels of WT and mutated α_2_δ subunits, whole-cell lysates (WCLs) from tsA201 cells transfected with 2HA-tagged α_2_δ were prepared and immunoblotted. At 48 h after transfection, cells were rinsed with phosphate-buffered saline (PBS), harvested, and resuspended in 200 µL ice-cold Pierce™ IP lysis buffer (Thermo Scientific, Waltham, MA, USA) supplemented with protease inhibitor cocktail (Thermo Scientific). Cells were homogenized using a sonicator (UP200S; Hieschler, Teltow, Germany) and lysed on ice for 30 min. Lysates were then cleared by centrifugation at 13,000× *g* for 15 min and assayed for total protein concentration using a Pierce BCA-protein assay kit (Thermo Scientific). Proteins were resuspended in a sample buffer containing 50 mM Tris (pH 6.8), 1.5 mM Bromphenol blue, 2% SDS, and 8% Glycerol. To cleave the disulfide bonds, sample buffer was supplemented with 150 mM dithiothreitol (DTT). When indicated, lysates were de-glycosylated with PNGase-F (NEB) following the manufacturer’s instructions and under conditions identical to those of the reduced samples. All samples were incubated at 95 °C for 10 min prior to loading onto 10% polyacrylamide gels (TGX™ FastCast™ Acrylamide 10% Kit, BIO-RAD, Berkeley, CA, USA) together with Color Pre-stained Protein Standard (NEB). Proteins were separated using a running buffer containing 25 mM Tris, 190 mM glycine, and 0.1% SDS, pH 8.7 at 80 V for 30 min, and 140 V for another 1–1.5 h. Proteins were then transferred to PVDF membranes with 0.45 µm pore size (Cytiva, Marlborough, MA, USA) in transfer buffer containing 80% running buffer and 20% ethanol, and using Trans-Blot Turbo (BIO-RAD) at 25 V (1.5 A) for 35 min at RT. After protein transfer, membranes were blocked with 5% milk in Tris-buffered saline (10 mM Tris-Base and 0.85% NaCl) with 0.3% tween (TBS-T) for 30 min at room temperature, followed by incubation with the indicated primary antibody in blocking buffer overnight at 4 °C. The following day, the membranes were washed 3 times with TBS-T and incubated with secondary antibodies coupled to Horseradish Peroxidase (HRP) for 60 min. Membranes were washed again three times for 10 min with TBST-T. The signal was obtained by HRP reaction with SuperSignal™ West Pico PLUS Chemiluminescent Substrate (Thermo Scientific), and membranes were scanned for protein detection with ChemiDoc Imaging System (BIO-RAD). Protein quantification was performed with Image Studio Light 6.0 and Microsoft Excel. The signal intensity of 2HA-α_2_δ was normalized to the signal intensity of the loading internal control α-Tubulin or GFP signals. Then, the relative signal intensities were normalized to the corresponding WT 2HA-α_2_δ relative signal intensity for each experiment separately.

### 4.8. Electrophysiology

Ca_V_2.1, Ca_V_1.2, and Ca_V_1.3 currents were measured using the whole-cell patch-clamp technique in voltage-clamp mode as described previously [60]. Patch pipettes were generated by pulling borosilicate glass capillaries (Sutter Instrument, Novato, CA, USA) with a micropipette puller (P-97, Sutter Instrument). Pipettes were then fire-polished using a MF-830 microforge (Narishige Co., Tokyo, Japan) and had resistances of 2−3.5 MΩ when filled with internal solution containing (in mM) 135 cesium chloride (CsCl), 10 Cs-EGTA (Carl Roth), 1 magnesium chloride (MgCl_2_), 10 HEPES (Carl Roth), and 4 Na_2_ATP (Sigma), adjusted to pH 7.3 with 1 M cesium hydroxide (CsOH). For measuring Ca^2+^ currents of Ca_V_1.2 and Ca_V_1.3 channels, a bath solution with 15 mM Ca^2+^ was used containing (in mM) 15 calcium chloride (CaCl_2_, Carl Roth), 150 Choline-Cl (Sigma), 1 MgCl_2_, and 10 HEPES, adjusted to pH 7.3 with 1 M CsOH. While measuring Ca^2+^ currents through Ca_V_2.1 channels, a bath solution with 2 mM Ca^2+^ was used containing (in mM) 2 CaCl_2_, 170 Choline-Cl, 1 MgCl_2_, and 10 HEPES, adjusted to pH 7.3 with 1 M CsOH. Whole-cell patch-clamp recordings were performed at room temperature (20–23 °C) using an EPC 10 amplifier controlled by Patch Master Software (HEKA Elektronik, Reutlingen, Germany). Current–voltage (I–V) relationships were measured by applying 50 ms depolarizing square pulses to various test potentials (from −80 mV to +90 mV) in steps of 5 mV, and holding potential (HP) was set to −80 mV. Linear leak and capacitive currents were digitally subtracted with a P/4 prepulse protocol. I–V curves were fitted according to a Boltzmann equation:

$$I=G_{MAX}\times \left(V-V_{rev}\right)/\left(1+e^{-\left(V-V_{0.5}\right)/k}\right)$$

I is the peak current, G_MAX_ is the maximum conductance, V is the test potential, V_rev_ is the extrapolated reversal potential, V_0_._5_ is the half-maximal activation voltage, and k is the slope factor. Series resistance was compensated by 80–95% and recordings were accepted for analysis when the I_peak_ was greater than 100 pA and smaller than 3 nA in size. Voltages were not corrected for the liquid junction potential (−9 mV).

### 4.9. Sequence Alignment

Protein sequences of α_2_δ from different species were obtained from the UniProt database and aligned with the Clustal Omega (EMBL-EBI) multiple sequence alignment program [78]. UniProt accessions of α_2_δ-1: P54289 for humans, O08532 for mice, P54290 for rats, A0A6D2W5R1 for chimpanzees, F1N7F9 for bovine, and A0A8M6Z241 for zebrafish. UniProt accessions of α_2_δ-3: Q8IZS8 for human, Q9Z1L5 for mouse, Q8CFG5 for rat, A0A2J8P813 for chimpanzee, A0A3Q1LU76 for bovine, and A0A8M2BK91 for zebrafish.

### 4.10. Structural Modelling

The structural homology modeling of human α_2_δ-3 protein was obtained by superposing the predicted AlphaFold protein structure [79,80] onto the published cryo-EM structure of human α_2_δ-1 subunit within the Ca_V_2.2 complex (PDB code: 7MIY) using PyMOL (The PyMOL Molecular Graphics System, version 2.3.2. Schrödinger, LLC, New York, NY, USA). Because the predicted structure of α_2_δ-3 was similar to α_2_δ-1 subunit, and A275 in α_2_δ-3 corresponds in α_2_δ-1 to a hydrophobic residue, isoleucine (I272), with similar properties to alanine, α_2_δ-1 structure was used to indicate the location of A275 and R351 residues (confer Figure 1).

### 4.11. Statistical Analysis and Experimental Design

Three to four independent hippocampal culture preparations were analyzed per experiment, and details on cell or bouton numbers are given in the respective figure legends. Electrophysiological recordings in tsA201 cells were obtained from three to four independent experiments (i.e., cell passage and transfection), and the number of recordings is given in the respective figure legends. Where possible, investigators were blinded during experiments and analyses. The distribution of all acquired data was visually assessed using the frequency distribution function of GraphPadPrism 9. All data are shown as mean ± SEM. No test for outliers was conducted, and all data points were included in the analysis. Significance levels (*p*-values) of statistical tests (one-way ANOVA or unpaired t-test) and post hoc analysis (Tukey’s multiple comparison test) are presented in the respective figure legends. The structural model in Figure 1 was generated with PyMOL (The PyMOL Molecular Graphics System, Version 2.3.2). Data, graphs, and figures were organized, analyzed, and assembled using MicrosoftExcel, GraphPadPrism 6, SigmaPlot (Systat Software), Adobe PhotoshopCS6, and Affinity Photo. Data contained within the article and raw data presented in this study are available upon request.

## Figures and Tables

**Table 1:** **ASD-associated α_2_δ mutations**. List of human α_2_δ mutations reported in patients with autism spectrum disorder. According to the SFARI-Gene database, which comprises a list of approximately 800 genes with identified potential autism-causing mutations and ranks them based on the evidence supporting their link to ASD. CACNA2D1 is ranked as a strong candidate gene of autism with a score of 2, while CACNA2D3 has very high confidence with a score of 1 (SFARI-Gene database of the Simons Foundation (https://gene.sfari.org, accessed on 15 October 2024 [25]). Allele frequency was determined using the Genome Aggregation Database (gnomAD, [28]) which comprises more than 800,000 control individuals. Evidence for associations between these genetic variants and human diseases was not available in the ClinVar database [29]. In silico pathogenicity predictions were made using PolyPhen2 (Polymorphism Phenotyping v2, benign variants: score ≤ 0.446; possibly damaging variants: 0.446 < score ≤ 0.908; probably damaging variants: score > 0.908 [30]). NA indicates not available (inheritance) or not applicable (PolyPhen-2).

Gene	Sequence Changes	Inheritance	gnomAD v4 Allele Frequency	PolyPhen-2	Reference
**CACNA2D1** (SFARI-Gene Score 2)	c.832del/p.Glu278LysfsTer3	De novo	Not reported	NA	[31]
	**c.1052G>C**/**p.Arg351Thr**	De novo	Single heterozygote allele	Probably damaging	[26]
	c.2903C>A/p.Ser968Tyr	NA	<0.0001, no homozygotes	Benign	[32]
	c.3101C>T/p.Ala1034Val	De novo	<0.0001, one homozygote	Probably damaging	[31]
**CACNA2D3** (SFARI-Gene Score 1)	c.2057-2A>G splice-site	De novo	Not reported	NA	[6]
	c.613C>G/p.Arg205Gly	NA	<0.001, no homozygotes	Probably damaging	[4]
	**c.823G>A**/**p.Ala275Thr**	Paternal	<0.00001, no homozygotes	Probably damaging	[27]
	c.1018A>G/p.Ile340Val	Maternal	<0.001, no homozygotes	Benign	[33]
	c.1163G>A/p.Arg388Gln	Maternal	<0.00001, no homozygotes	Probably damaging	[34]
	c.1195C>T/p.Arg399Ter	De novo	<0.00001, no homozygotes	NA	[35]
	c.1360G>A/p.Glu454Lys	Maternal	<0.0001, no homozygotes	Probably damaging	[33]
	c.1522G>T/p.Glu508Ter	De novo	Not reported	NA	[4]
	c.1733G>A/p.Arg578Gln	NA	<0.0001, no homozygotes	Probably damaging	[4]
	c.1993C>T/p.Arg665Cys	NA	<0.0001, no homozygotes	Probably damaging	[4]
	c.2092G>A/p.Ala698Thr	NA	<0.001, no homozygotes	Probably damaging	[4]
	c.2093C>T/p.Ala698Val	Maternal	<0.0001, no homozygotes	Probably damaging	[36]
	c.2164G>A/p.Gly722Ser	Paternal	Single heterozygote allele	Probably damaging	[34]
	c.2191C>G/p.Arg731Gly	NA	<0.00001, no homozygotes	Probably damaging	[4]
	c.2195C>G/p.Thr732Arg	De novo	Single heterozygote allele	Possibly damaging	[34]
	c.2266G>A/p.Asp756Asn	NA	<0.00001, no homozygotes	Probably damaging	[4]
	c.2318C>T/p.Ala773Val	Maternal	<0.00001, no homozygotes	Probably damaging	[27]
	c.2749G>A/p.Ala917Thr	Maternal	<0.0001, no homozygotes	Possibly damaging	[36]

**Table 2:** **Current properties of Ca_V_2.1 in tsA201 cells**.

	Ca_V_2.1/β_4_/α_2_δ-1			Ca_V_2.1/β_4_/α_2_δ-3		
	Control	WT	R351T	Control	WT	A275T
**Current density (pA/pF)**	−14.7 ± 1.9	−68.6 ± 8.3	−70.2 ± 5.5	−10.1 ± 1.2	−70.9 ± 10.8	−63.2 ± 8.5
**V_1/2_ (mV)**	−3.1 ± 0.5	−3.2 ± 0.3	−3.5 ± 0.4	−3.6 ± 0.6	−4.9 ± 0.3	−5.3 ± 0.4
**V_rev_ (mV)**	44.9 ± 0.8	44.0 ± 0.4	43.9 ± 0.4	46.0 ± 1.1	44.3 ± 0.4	45.1 ± 0.7
***n***	10	27	33	6	17	19

All values are presented as mean ± SEM and were obtained from three independent experiments. V_1/2_ and V_rev_ parameters were obtained by fitting the I–V curves to a Boltzmann function. V_1/2_, half-maximal activation potential; V_rev_, reversal potential; *n*, number of recordings. For statistics see Figure 6.

**Table 3:** **Current properties of Ca_V_1.3 in tsA201 cells**.

	Ca_V_1.3/β_3_/α_2_δ-1			Ca_V_1.3/β_3_/α_2_δ-3		
	Control	WT	R351T	Control	WT	A275T
**Current density (pA/pF)**	−10.1 ± 1.5	−7.9 ± 10.6	−57.3 ± 11.04	−14.3 ± 1.3	−81.3 ± 13.6	−80.2 ± 13.8
**V_1/2_ (mV)**	11.9 ± 1.3	4.8 ± 0.6	4.7 ± 0.6	14.7 ± 1.0	4.2 ± 0.6	4.3 ± 0.8
**V_rev_ (mV)**	81.2 ± 1.1	72.5 ± 0.9	72.9 ± 1.0	73.7 ± 2.5	71.9 ± 0.7	72.2 ± 0.7
***n***	10	23	22	6	15	16

All values are presented as mean ± SEM and were obtained from more than two independent experiments. V_1/2_ and V_rev_ parameters were obtained by fitting the I–V curves to a Boltzmann function. V_1/2_, half-maximal activation potential; V_rev_, reversal potential; *n*, number of recordings. For statistics see Figure 7.

**Table 4:** **Current properties of Ca_V_1.2 in tsA201 cells**.

	Ca_V_1.2/β_1_/α_2_δ-1		
	Control	WT	R351T
**Current density (pA/pF)**	−32.5 ± 3.1	−110.7 ± 12.5	−114.7 ± 13.1
**V_1/2_ (mV)**	17.7 ± 1.0	8.4 ± 0.6	9.1 ± 0.9
**V_rev_ (mV)**	83.9 ± 1.2	75.7 ± 0.9	74.9 ± 1.8
***n***	18	22	24

All values are presented as mean ± SEM and were obtained from three independent experiments. V_1/2_ and V_rev_ parameters were obtained by fitting the I–V curves to a Boltzmann function. V_1/2_, half-maximal activation potential; V_rev_, reversal potential; *n*, number of recordings. For statistics, see Figure 8.

**Table 5:** **List of antibodies used in this study**. Information on primary and secondary antibodies used in western blot and immunocytochemistry experiments.

Antibody	Species	Dilution	Source
Anti-HA	Rat, clone 3F10	1:100 (Live/A594) 1:1000 (permeabilized/A594)	Roche (cat. no. 11867423001, RRID: AB_390918)
Anti-HA	Mouse, clone 5B1D10	1:1000 (WB)	Thermo Fisher Scientific (cat. no. 32-6700, RRID: AB_2533092)
Anti-GABA_A_R_β2/3_	Mouse, clone bd17	1:500 (A594)	Millipore (cat. no. MAB341, RRID: AB_2109419)
Anti-synapsin1	Mouse, clone 46.1	1:500 (A350)	Synaptic Systems (cat. no. 106 011, RRID: AB_2619772)
Anti-vGLUT1	Rabbit, polyclonal	1:2000 (A350)	Synaptic Systems (cat. no. 135 002, RRID: AB_2315546)
Anti-α Tubulin	Mouse, clone DM1A	1:1000	Abcam (cat. no. ab7291, PRID: AB_2241126)
Anti-GFP	Mouse, clone 3E6	1:10,000	Thermo Fisher Scientific (cat. no. A-11120, PRID: AB_221568)
Alexa Fluor 350	Goat anti-rabbit	1:500	Thermo Fisher Scientific (cat. no. A-21068, RRID: AB_2535729)
	Goat anti-mouse	1:500	Thermo Fisher Scientific (cat. no. A-21049, RRID: AB_2535717)
Alexa Fluor 594	Goat anti-mouse	1:4000	Thermo Fisher Scientific (cat. no. A-11032, RRID: AB_2534091)
	Goat anti-rat	1:4000	Thermo Fisher Scientific (cat. no. A-11007, RRID: AB_10561522)
Secondary antibody coupled to HRP	Goat anti-mouse IgG [H+L]	1:20,000	Thermo Fisher Scientific (cat. no. G21040, RRID: AB_2536527)

## Data Availability

Data are contained within the article.
